# MabsBase: A *Mycobacterium abscessus* Genome and Annotation Database

**DOI:** 10.1371/journal.pone.0062443

**Published:** 2013-04-29

**Authors:** Hamed Heydari, Wei Yee Wee, Naline Lokanathan, Ranjeev Hari, Aini Mohamed Yusoff, Ching Yew Beh, Amir Hessam Yazdi, Guat Jah Wong, Yun Fong Ngeow, Siew Woh Choo

**Affiliations:** 1 Department of Medical Microbiology, Faculty of Medicine, University of Malaya, Kuala Lumpur, Malaysia; 2 Dental Research and Training Unit, Faculty of Dentistry, University of Malaya, Kuala Lumpur, Malaysia; 3 Department of Software Engineering, Faculty of Computer Science and Information Technology, University of Malaya, Kuala Lumpur, Malaysia; 4 Department of Computer System & Technology, Faculty of Computer Science and Information Technology, University of Malaya, Kuala Lumpur, Malaysia; Kyushu Institute of Technology, Japan

## Abstract

*Mycobacterium abscessus* is a rapidly growing non-tuberculous mycobacterial species that has been associated with a wide spectrum of human infections. As the classification and biology of this organism is still not well understood, comparative genomic analysis on members of this species may provide further insights on their taxonomy, phylogeny, pathogenicity and other information that may contribute to better management of infections. The MabsBase described in this paper is a user-friendly database providing access to whole-genome sequences of newly discovered *M. abscessus* strains as well as resources for whole-genome annotations and computational predictions, to support the expanding scientific community interested in *M. abscessus* research. The MabsBase is freely available at http://mabscessus.um.edu.my.

## Introduction

*Mycobacterium abscessus* is a rapidly growing non-tuberculous mycobacterium (NTM) first discovered by Moore and Frerichs in 1953 when it was isolated from a knee abscess [1]. This bacterium was considered a member of the *Mycobacterium chelonae* group until 1992 when it was re-classified as a separate species [2], [3]. It has since become known as an important opportunistic pathogen in humans, being associated with a wide spectrum of superficial skin and soft tissue infections as well as serious disseminated infections in immunocompromised patients [4], [5]. It is particularly prominent as a pathogen in broncho-pulmonary infections in patients with cystic fibrosis and chronic lung disorders [4], [6].

*M. abscessus* was recently further divided into three subspecies, namely *M. abscessus* sensu stricto, *M. bolletii* and *M. massiliense,* on the basis of their genetic composition. They are known to differ from one another in their *rpoB*, *hsp65* and other housekeeping genes and in their susceptibility to antibiotics [7], [8]. Further comparative genomic analysis of these subspecies will give us a better understanding of their genetic and biological properties.

*M. abscessus* sensu stricto was first sequenced and annotated by Ripoll and co-workers under the strain name *M. abscessus* CIP 104536T [9]. Since then, more *M. abscessus* subspecies have been sequenced and increasing numbers of these genomes are being lodged in the NCBI database. We set up the MabsBase to facilitate comparative genomic analysis between strains as well as to systematically assign their taxonomy based on important genes. We also aim to provide resources for whole-genome annotations and computational predictions specifically designed to support the expanding *M. abscessus* research community, with whom we hope to collectively gather all information on existing and new strains of *M. abscessus* into one database so that interested parties can gain access to the information, genomes, sequences, and annotations in the database. Here we describe the overview of the MabsBase.

## Methods

### Overview

This database currently comprises 40 *M. abscessus* genomes obtained from Genbank. Twelve of these were sequenced by our group using the Illumina Genome Analyzer 2X platform [10], [11], [12], [23]–[28]. This sequencing platform uses short read technology to generate very high outputs and a large number of reads per run at a competitive cost. Its high coverage is crucial for the de-novo assembly of large genomes. Many bioinformatics tools and software have been developed to be compatible with the Illumina-based data format which simplifies the downstream analysis. The GA 2X technology is a widely adopted next-generation system that has been successfully used for the sequencing of many organisms [10], [11]. As our study at the University of Malaya involved only genomic analysis of isolates obtained from routine cultures and no patient information is divulged, it was considered unnecessary to apply for ethical approval by the University's Medical Ethics Committee Standard Operating Procedures (http://www.ummc.edu.my/index.php/2011-09-28-08-46-26/2011-10-03-03-14-40/158-ummc-medical-ethics).

All 40 genome sequences were annotated by using the Rapid Annotation using Subsystem Technology (RAST) pipeline [12]. This pipeline is a fully automated annotation engine for complete or draft archaeal and bacterial genomes. RAST is able to identify various important components in a genome such as protein encoding genes, rRNA and tRNA, pseudogenes, gene functions and subsystems prediction. The pipeline then utilizes this information to construct the metabolic network and generate user-friendly, downloadable results. Protein assignments in the pipeline are based on functional properties, i.e. proteins are predicted according to the closely-relatedness within the subsystems in FIGfams database [13]. All annotations including genes, RNAs and predicted protein functions of *M. abscessus* strains are stored in our mySQL database. The *M. abscessus* strain ATCC 19977 is used as the reference genome for the determination of genome coverage and identity of other *M. abscessus* strains [9].

### Database Organization and Features

**Figure 1:**
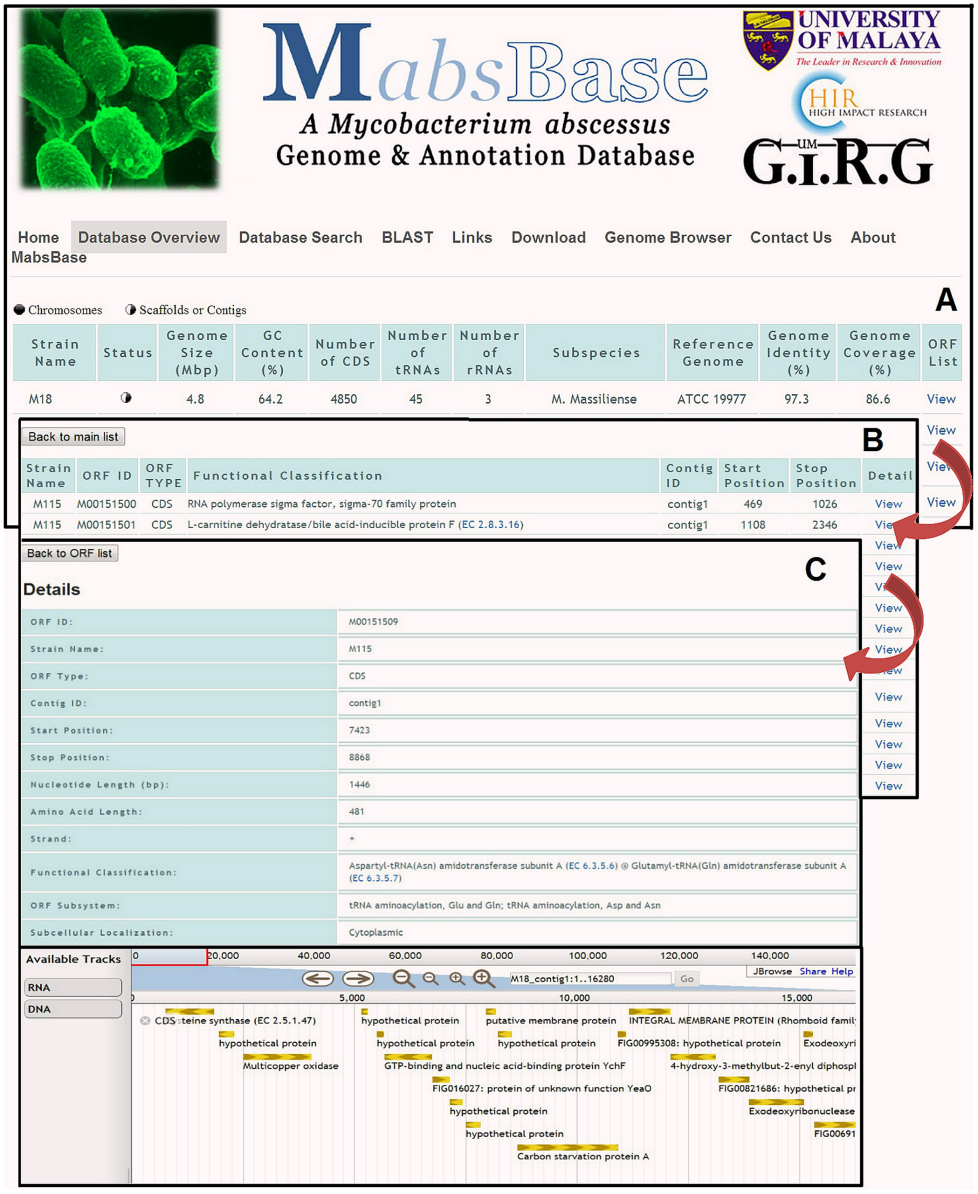
Overview of MabsBase. (A) The database overview which displays the main list of all strains and information such as genome size, genome identity and coverage etc., organized in columns. (B) ORF list of a specific strain. (C) Detailed information of an ORF with visualization in JBrowse.

**Figure 2:**
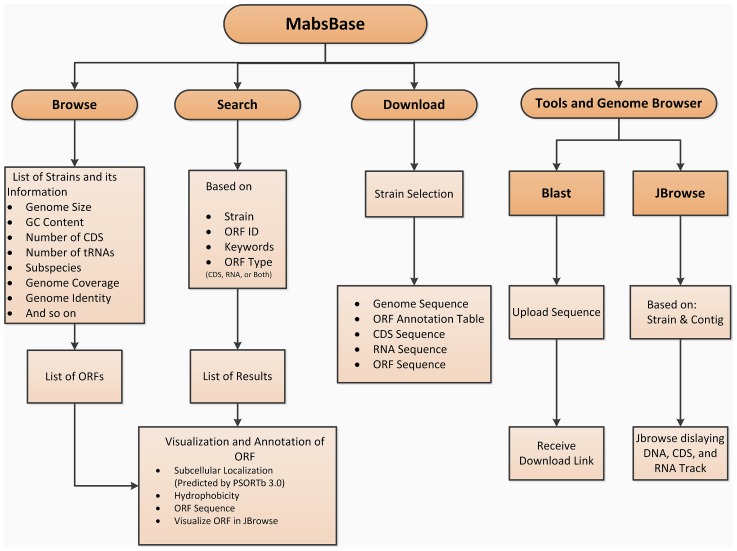
A diagram showing the overview of the functionalities in MabsBase.

The MabsBase is user-friendly with straightforward applications and tools. The database overview tabulates the main list of *M. abscessus* strains and related information such as the genome size, number of coding sequences, number of tRNAs and rRNAs, genome identity and coverage, GC content and predicted subspecies type, organized in columns. The ORF (Open Reading Frame) list of each strain is accessible by following the ORF link of the desired strain. Detailed information of an ORF such as its ORF ID and type, function or subsystem classification and its start and stop positions are available with built-in Jbrowse [14] to enable users to further visualize the ORF within a particular contig (Figure 1). Users can perform a direct search for information under the database search tab by applying query filters either strain type, ORF ID or relevant keywords, without having to screen through the full list of available strains. MabsBase has a built-in BLAST tool for sequence search in the database. BLAST [15] compares the region of similarity between nucleotide or amino acid sequences resulting in a ranked list of alignment scores known as hits which are then evaluated in various statistics such as query coverage and E-value. The custom download menu of this database allows users to download strain specific data such as genome assembly, ORF annotations, coding sequence (CDS) or RNA sequences. The overview of the functionalities in Mabsbase is shown in Figure 2.

### MabsBase Development

The MabsBase is developed using the 3-tier software development architecture which provides a high level of performance and scalability. PHP is the scripting language used to build this website and the object-oriented concepts such as encapsulation, inheritance, and polymorphism were applied to achieve a high level of maintainability and extensibility which is crucial in terms of software evolution especially for future work. MySQL relational database is employed to store information such as strain and feature details, annotation tables, and sequences. By using the stored procedures and views in MySQL database we tried to achieve a higher level of performance and reliability. The BLAST service is provided by utilizing the application server in order to perform the process for different users in parallel, and last but not least, we applied data encryption, input validation, privilege sets, and other secure programming techniques to attain a higher level of security in order to increase the reliability which may impact on the level of availability of the website.

### Bioinformatics Tools Used in Analysis

Subsequent to the advent of next-generation sequencing technologies, the number of incomplete or draft genomes released increased rapidly, surpassing the number of completed genomes released. An effective approach to make meaningful analysis of this enormous amount of data is to compare the incomplete genomes against the reference genome. We used the rapid, whole-genome aligning system of MUMmer version 3.0 [16] to align the incomplete genomes of new *M. abscessus* strains against the reference genome of *M. abscessus* strain ATCC 19977, in order to obtain information such as genome coverage and the identity of each draft genome sequence relative to the reference genome. The NUCmer program in the MUMmer system is capable of handling 100 to 1,000 contigs generated by shortgun sequencing and aligning them to another set of contigs or another genome. The PROmer program, on the other hand, can generate alignments based on the six-frame translations of both input sequences, in cases where the query sequences are too divergent for DNA sequence alignment. Both the PROmer and NUCmer can, therefore, efficiently align the incomplete sequences onto the complete genome, constructing reliable sequences while eliminating repetitive elements or regions.

To predict the subcelluar localization of each putative protein, we used the latest PSORTb version 3.0 (Figure 2) with new improvements for protein subcellular localization prediction [17]. This tool is developed and maintained by the Brinkman lab of Simon Fraser University in British Columbia, Canada. PSORTb is a tool commonly used for genome analysis and annotation as protein subcellular localization may predict the functional elements in a bacterium. The latest PSORTb version 3.0 has a significant increase in the recall of predictions and proteome prediction coverage at high precision with improved features such as options for the prediction of archaeal and atypical prokaryotic proteins, prediction capability for ambiguous Gram-positive or Gram-negative bacterial proteins and refined sub-categories localization. Incorporating this precise prediction tool allows us to perform a quick and inexpensive computational prediction in this database.

## Results and Discussion

### *Mycobacterium abscessus* Subspecies Classification Using Core Genome SNPs

**Figure 3:**
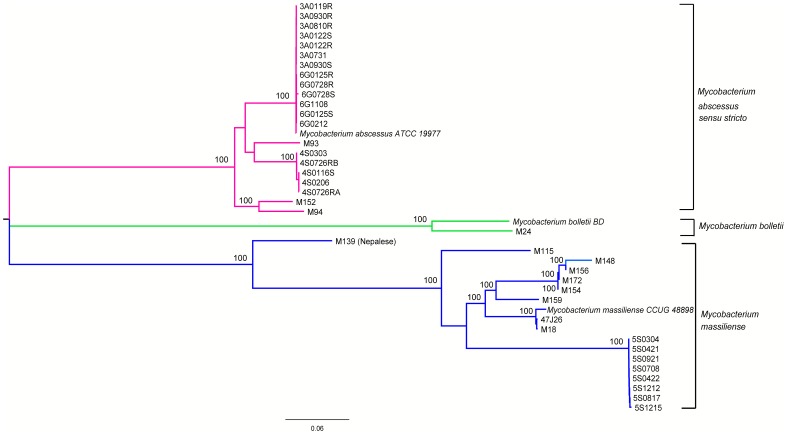
Core genome SNPs-based phylogenetic tree. All isolates were clustered into three distinct groups. Countries where the sample collection originated are indicated in parentheses. Concatenated core genome SNPs sequences were aligned and phylogenetic inferences obtained using the maximum-likelihood method within the MEGA 5.1 software. Numbers at the nodes are percentages of bootstrap values obtained by repeating the analysis 1,000 times to generate a majority consensus tree. The scale bar represents a 6% sequence difference.

We examined the classification of *M. abscessus* subspecies using SNP information contained in their core genomes. Using Panseq [21], we identified the core genomes by aligning all 40 genome sequences included in this study, two subspecies reference genomes (*M. massiliense CCUG 48898* and *M. bolletii BD*). All SNPs in each core genome were extracted and concatenated into a supersequence. The concatenated sequences from all strains were aligned and a phylogenetic tree was plotted using MEGA 5.1 software [22]. The resulting tree (Figure 3) showed all strains clustering with the subspecies reference strains into three major groups corresponding to the three currently accepted *M. abscessus* subspecies. This tree can be used for the subspecies identification of new *M. abscessus* strains. The details of the classification method will be described in our main paper (*Tan et al*, manuscript in preparation).

### Future Developments

MabsBase will be updated from time to time as more genome annotations and genomic sequences of *M. abscessus* become available. Identifying variations in these genome assemblies would lead to a better understanding of *M. abscessus* diversity and evolution, and possibly to the pathogenicity and drug resistance of this pathogen if patient information is available. Further analyses on these data are ongoing in our research group and will be incorporated into MabsBase from time to time. For instance, we are planning to incorporate RNA sequences into the genome browser for the validation of predicted genes. To accelerate the development of this Mabsbase for the use of the scientific community, we encourage other research groups to email us at girg@um.edu.my if they would like to share annotations, curations and related datasets with us. Suggestions on improving this database and requests for additional functions are also welcome.
