# Correction: MabsBase: A *Mycobacterium abscessus* Genome and Annotation Database

**DOI:** 10.1371/annotation/0114fde1-da1c-4661-a233-e50779aa7d3a

**Published:** 2013-05-02

**Authors:** Hamed Heydari, Wei Yee Wee, Naline Lokanathan, Ranjeev Hari, Aini Mohamed Yusoff, Ching Yew Beh, Amir Hessam Yazdi, Guat Jah Wong, Yun Fong Ngeow, Siew Woh Choo

There was an error in the funding statement. The correct funding information is:

This research is supported by UM High Impact Research Grant UM-MOHE UM.C/HIR/MOHE/08 from the Ministry of Higher Education Malaysia and High Impact Research Grant UM.C/625/1/HIR/003/1 from the University of Malaya. The funders had no role in study design, data collection and analysis, decision to publish, or preparation of the manuscript. 

